# PRIMARY TOTAL HIP ARTHROPLASTIES UNDER BRAZILIAN PUBLIC HEALTH SYSTEM (2012-2021)

**DOI:** 10.1590/1413-785220233103e268117

**Published:** 2023-09-22

**Authors:** Tarcísio Marconi Novaes Torres, Brenna Kathleen Martins, Alan Almeida da Silva, Carlos Alberto Almeida de Assunção, Enilton de Santana Ribeiro de Mattos, Alex Guedes

**Affiliations:** 1 Universidade Federal da Bahia, Complexo Hospitalar Universitário Professor Edgard Santos, Programa de Residência Médica em Ortopedia e Traumatologia, Empresa Brasileira de Serviços Hospitalares, Salvador, BA, Brazil.; 2 Secretaria de Saúde do Estado da Bahia, Hospital Regional de Santo Antonio de Jesus, Programa de Residência Médica em Ortopedia e Traumatologia do Santo Antonio de Jesus, BA, Brazil.; 3 Universidade Federal da Bahia, Unidade do Sistema Neuro-Músculo-Esquelético, Empresa Brasileira de Serviços Hospitalares, Salvador, BA, Brazil.; 4 Universidade Federal da Bahia, Faculdade de Medicina da Bahia, Departamento de Cirurgia Experimental e Especialidades Cirúrgicas, Salvador, BA, Brazil.

**Keywords:** Arthroplasty, Replacement, Hip, Hip Fractures, Hospital Costs, Length of Stay, Mortality, Regional Health Planning, Artroplastia de Quadril, Fraturas do Quadril, Custos Hospitalares, Tempo de Internação, Mortalidade, Regionalização da Saúde

## Abstract

**Objectives:**

To describe the regional distribution of hospital admission authorizations (HAA), hospitalization costs (HC), the average length of stay (LOS), and mortality rates (MR) related to primary total hip arthroplasties (THA) funded by the Brazilian Health Unic System (SUS) from 2012 to 2021.

**Methods:**

Descriptive cross-sectional study using secondary data of public domain obtained from the Department of Informatics of SUS (DATASUS) database website.

**Results:**

A total of 125,463 HAA were released with HC of 552,218,181.04 BRL in the evaluated period. The average LOS was of 6.8 days. MR was 1.62%.

**Conclusion:**

The regional distribution of HAA was 65,756 (52%) in the Southeast; 33,837 (27%) in the South; 14,882 (12%) in the Northeast; 9,364 (8%) in Midwest; and 1,624 (1%) in North - in 2020 there was a sharp decrease of the released HAA, probably due to the COVID-19 pandemic. HC was 293,474,673.20 BRL in the Southeast; 144,794,843.11 BRL in the South; 61,751,644.36 BRL in the Northeast; 45,724,353.80 BRL in the Midwest; and 6,472,666.57 BRL in the North. The average LOS was 6.7 in the Southeast; 5.3 in the South; 9.2 in the Northeast; 7.6 in the Midwest; and, 13.6 in the North. MR was as follows: Southeast=1.88%; South=1.07%; Northeast=1.83%; Midwest=1.44%; and North=1.47%. ***Evidence Level III; Retrospective Comparative Study*** .

## RESUMO

**Objetivos:**

Descrever a distribuição regional das autorizações de internação hospitalar (AIH), custos de internação (CI), tempo médio de permanência (TMP) e taxa de mortalidade (TM) relacionados às artroplastias totais de quadril (ATQ) primárias financiadas pelo Sistema Único de Saúde (SUS) de 2012 a 2021.

**Métodos:**

Estudo transversal descritivo utilizando dados secundários de domínio público obtidos no site do banco de dados do Departamento de Informática do SUS (DATASUS). Resultados: Foram liberadas 125.463 AIH com CI de R$ 552.218.181,04 no período avaliado. O TMP foi de 6,8 dias. A TM foi de 1,62%.

**Conclusões:**

A distribuição regional de AIH foi de 65.756 (52%) no Sudeste; 33.837 (27%) no Sul; 14.882 (12%) no Nordeste; 9.364 (8%) no Centro-Oeste; e, 1.624 (1%) no Norte - em 2020 houve queda acentuada das AIH liberadas, provavelmente devido à pandemia COVID-19. Os CI foram de R$ 293.474.673,20 no Sudeste; R$ 144.794.843,11 no Sul; R$ 61.751.644,36 no Nordeste; R$ 45.724.353,80 no Centro-Oeste; e R$ 6.472.666,57 no Norte. O TMP foi de 6,7 no Sudeste; 5,3 no Sul; 9,2 no Nordeste; 7,6 no Centro-Oeste; e 13,6 no Norte. A TM foi como se segue: Sudeste=1,88%; Sul=1,07%; Nordeste=1,83%; Centro-Oeste=1,44%; e, Norte=1,47%. ***Nível de Evidência III; Estudo Retrospectivo Comparativo*** .

## INTRODUCTION

Total hip arthroplasty (THA) is one of the most performed orthopedic surgical procedures in the world and considerably improves the patients’ quality of life, besides presenting a relatively short recovery period. Jones et al. (2000)^1^ pointed out to improvement in pain and functional status in more than 75% of cases, with patient's satisfaction rate reaching 91%. The main indications for THA in the elderly are advanced hip osteoarthritis (OA) and femoral neck fractures (FNF).

OA is one of the main degenerative diseases affecting the elderly population - a chronic and disabling condition, associated with pain, stiffness and, in the most severe cases, deformities. In Brazil,OA is the most prevalent musculoskeletal disease, affecting 4% of the population, being associated with falls, depression and obesity.^2^ The treatment is initially symptomatic, focused on the approach of pain and function preservation, based on the use of analgesics, anti-inflammatory drugs, weak opioids and physiotherapy. In more advanced cases, with greater joint involvement and pain worsening, it is necessary to replace the affected joint.

Hip fractures are among the most common lesions treated by orthopedists and especially prevalent in the geriatric population. In 2014, more than 320,000 hip fractures were treated in emergency rooms in the United States (US), most of them in women aged 65 and over. Each year, more than a third of adults aged 65 and over fall. The higher number of falls, combined with the higher prevalence of osteoporosis, makes the geriatric population particularly susceptible to fractures. Hip fractures reduce patient independence and mobility and are associated with increased mortality risk. There are several treatment options for FNF; non-deviated fractures can be treated by internal in situ fixation with screws, although several studies demonstrate that this approach is not ideal, especially in the elderly population, being preferably to perform hip arthroplasty, especially the partial (PHA) one. THA has historically been reserved for younger and more active patients with a history of hip OA; many studies, however, have shown that the functional results of THA are superior of those of PHA in the treatment of FNF.^3^

Moreover, THA can be divided into two large groups: cemented and not cemented. The first is based on the use of polymethylmethacrylate (PMMA) to fix the prosthetic components; this method has historically been associated with a high rate of aseptic loosening, demanding studies to improve the quality of the PMMA, in addition to the development of other fixation methods,^4^ emerging the concept of non-cemented prostheses, which fixation is based on press fit and osteointegration potential, presented and widely disseminated in the 1970s and 1980s, with the objective of improving of THA durability, avoiding loosening and bone destruction; however, it was found that non-cemented prostheses have loosening rates of 1.3% to 9% in femoral component and from 3% to 15% in acetabular component.^4^

Data on the regional distribution of performed primary THA in Brazil are scarce,^5^ even in view of the high prevalence of hip OA and FNF in this country. It becomes relevant to know about the volume of these procedures performed over the years, in addition to its associated costs, average length of stay (LOS) and mortality rates (MR), so that the health system can define appropriate strategies to deal with this reality, enabling improvement in the quality of care to the population affected by these conditions.

The purpose of this paper is to describe the regional distribution of hospital admission authorizations (HAA), hospitalization costs (HC), average LOS, and MR related to primary THA funded by the Brazilian Health Unic System (SUS) from 2012 to 2021.

## METHODS

This is a descriptive cross-sectional study dealing with the regional distribution (Midwest, North, Northeast, Southeast and South) of HAA, HC, average LOS, and MR related to primary THA funded by the Brazilian Health Unic System (SUS) from 1 January 2012 to 31 December 2021.

The secondary data were obtained from the database site of the Department of Informatics of the SUS (DATASUS), Ministry of Health. All hospitalizations for primary THA coded under the records 04.08.04.008-4 (primary cemented THA) and 04.08.04.009-2 (primary non-cemented/hybrid THA) of the Unified Table of Procedures, Medications, Orthotics, Prostheses and Synthesis Materials Management System (SIGTAP) were included.

For the calculations that required population data, we used the 2010 census, conducted by the Brazilian Institute of Geography and Statistics (IBGE). The Microsoft 365® Excel ® program was used to data tabulating and statistical calculations.

Due to the design of the study, in accordance with the National Health Council Resolution (CNS) no. 466/2012, no approval by the institutional research ethics committee was required, because we used secondary information from a public domain database.

## RESULTS

**Figure 1:**
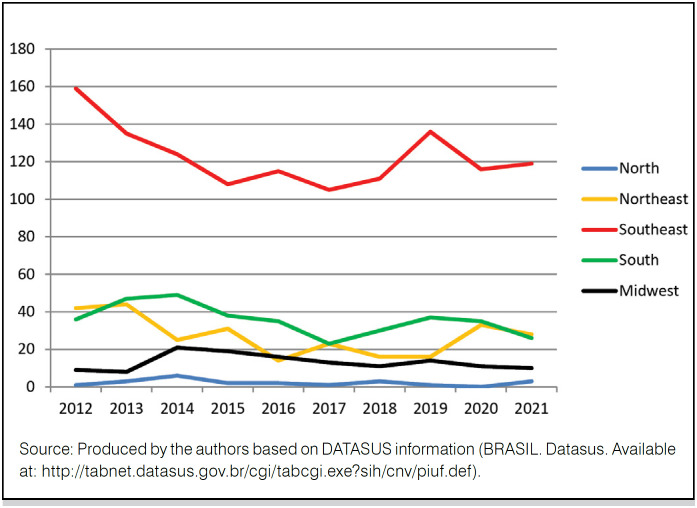
Absolute number of cemented and non-cemented/hybrid THA per year (2012-2021).

In the evaluated decade (2012-2021), the SUS funded 125,463 THA, of which 42,113 (33.6%) were cemented and 83,350 (66.4%) were non-cemented/hybrid ( Figure 1 ). The Southeast Region performed approximately 52% (65,756) of these procedures, followed by the South Region, with 27% (33,837); the Northeast Region, with 12% (14,882); the Midwest Region, with 8% (9,273); and the North Region, with 1% (1,624) ( Table 1 ).

**Table 1:** Hospital Admission Authorizations (HAA) Distribution for THA by Brazilian region per year (2012-2021).

Region	2012	2013	2014	2015	2016	2017	2018	2019	2020	2021	Total
North	113	164	208	213	146	196	194	139	121	130	1,624
Northeast	1,472	1,529	1,519	1,443	1,314	1,506	1,606	1,476	1,464	1,553	14,882
Southeast	6,254	6,817	6,735	6,628	6,751	6,683	7,251	7,577	5,352	5,708	65,756
South	3,041	3,357	3,510	3,771	3,630	3,359	3,916	4,134	2,616	2,503	33,837
Midwest	641	819	845	964	889	1,162	1,265	1181	801	797	9,364
Total	11,521	12,686	12,817	13,019	12,730	12,906	14,232	14,507	10,354	10,691	125,463

Source: Produced by the authors based on DATASUS information (BRASIL. Datasus. Available at: http://tabnet.datasus.gov.br/cgi/tabcgi.exe?sih/cnv/piuf.def ).

**Figure 2:**
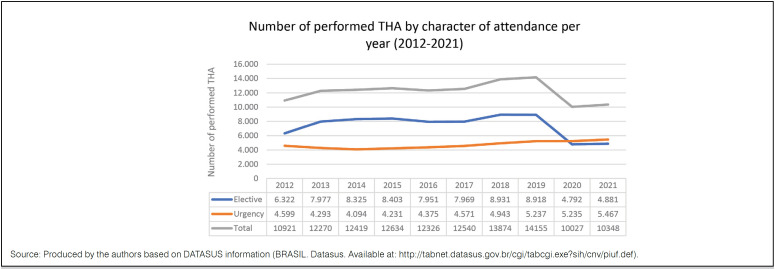
umber of performed THA by character of attendance per year (2012-2021).

Most of primary THA (59.36%) were performed as elective procedures, presenting an increase of 41.1% in number from 2012 to 2019; however, between 2019 and 2020, occurred an expressive fall (46.3%) of elective THA. The urgency procedures experienced a percentage increase of 13.9% between 2012 and 2019, remaining stable in number between 2019 and 2020. There was no information on hospitalization regimen in 3,949 of the performed procedures ( Figure 2 ).

The total expenditure made by the SUS for primary THA in the evaluated period was of 552,218,181.04 BRL. The Southeast region obtained the largest investment (293,474,673.20 BRL, 53.14% of the total amount spent), followed by the South, Northeast, Midwest and North Regions ( Table 2 ). Moreover, the mean value per hospitalization was of 4,394.92 BRL; the Midwest Region obtained the greater mean value per hospitalization (4,820.00 BRL) ( Table 3 ).

**Table 2:** Distribution of the total amount spent in BRL with hospitalizations for THA by Brazilian region per year (2012-2021).

Region	2012	2013	2014	2015	2016	2017	2018	2019	2020	2021	Total
North	274,827.13	552,218.37	871,571.68	933,070.22	452,995.56	707,935.75	914,676.08	682,713.52	565,458.13	517,200.13	6,472,666.57
Northeast	5,190,104.72	5,953,644.26	5,976,891.83	5,680,239.12	5,519,094.07	6,518,046.77	7,093,242.93	6,705,893.97	6,338,972.86	6,775,513.83	61,751,644.36
Southeast	22,995,168.75	29,154,816.93	29,448,373.28	30,093,439.86	29,329,746.77	29,677,185.98	34,432,053.73	36,530,191.69	25,071,105.81	26,742,590.40	293,474,673.20
South	10,771,406.54	13,890,234.17	14,561,727.83	15,890,478.00	15,200,258.03	14,157,902.47	17,364,872.23	19,180,027.15	12,088,572.61	11,689,364.08	144,794,843.11
Midwest	2,139,227.27	3,544,996.30	4,008,112.30	4,933,774.71	4,985,483.17	6,231,197.26	5,979,867.13	5,987,737.13	3,911,088.10	4,002,870,43	45,724,353.80
Total	41,370,734.41	53,095,910.03	54,866,676.92	57,531,001.91	55,487,577.60	57,292,268.23	65,784,712.10	69,086,563.46	47,975,197.51	49,727,538.87	552,218,181.04

Source: Produced by the authors based on DATASUS information (BRASIL. Datasus. Available at: http://tabnet.datasus.gov.br/cgi/tabcgi.exe?sih/cnv/piuf.def ).

**Table 3:** Average BRL value of hospital admissions for THA by Brazilian region per year (2012-2021).

Region	2012	2013	2014	2015	2016	2017	2018	2019	2020	2021	Average
North	2,432.10	3,367.19	4,190.25	4,380.61	3,102.71	3,611.92	4,714.83	4,911.61	4,673.21	4,420.51	3,980.49
Northeast	3,525.89	3,893.82	3,934.75	3,936.41	4,200.22	4,328.05	4,416.71	4,543.29	4,329.90	4,362.86	4,147.19
Southeast	3,676.87	4,276.78	4,372.44	4,540.35	4,344.50	4,440.70	4,748.59	4,821.19	4,684.44	4,685.11	4,459.10
South	3,542.06	4,137.69	4,148.64	4,213.86	4,187.40	4,214.92	4,434.34	4,639.58	4,621.01	4,670.14	4,280.96
Midwest	3,337.33	4,328.44	4,743.33	5,118.02	5,607.97	5,362.48	4,727.17	5,070.06	4,882.76	5,022.42	4,820.00
Average	3,590.90	4,185.39	4,280.77	4,419.00	4,358.80	4,439.20	4,622.31	4,762.29	4,633.49	4,657.01	4,394.92

Source: Produced by the authors based on DATASUS information (BRASIL. Datasus. Available at: http://tabnet.datasus.gov.br/cgi/tabcgi.exe?sih/cnv/piuf.def ).

The average LOS for primary THA in the evaluated period was of 6.8 days ( Table 4 ). The North Region presented the highest average LOS (13.6 days), while the South Region presented the lowest(5.3 days) for primary THA.

**Table 4:** Average length of stay (LOS) in days for THA by Brazilian region per year (2012-2021).

Region	2012	2013	2014	2015	2016	2017	2018	2019	2020	2021	Average
North	11,6	12,3	15,4	13,2	13,9	13,9	16,6	13,4	12,2	13,1	13,6
Northeast	9,1	9,5	9,2	9,7	10,5	9,2	9,2	8,6	9,1	7,8	9,2
Southeast	7,2	7	6,9	6,9	6,8	6,5	6,4	6,3	6,6	6,3	6,7
South	5,9	5,9	5,8	5,5	5,2	5,2	5	4,7	5	4,7	5,3
Midwest	9,9	8,7	7,6	7,2	7,6	6,9	8,2	7,1	6,4	6,1	7,6
Average	7,3	7,2	7,1	7	6,9	6,7	6,6	6,2	6,6	6,2	6,8

Source: Produced by the authors based on DATASUS information (BRASIL. Datasus. Available at: http://tabnet.datasus.gov.br/cgi/tabcgi.exe?sih/cnv/piuf.def ).

**Figure 3:**
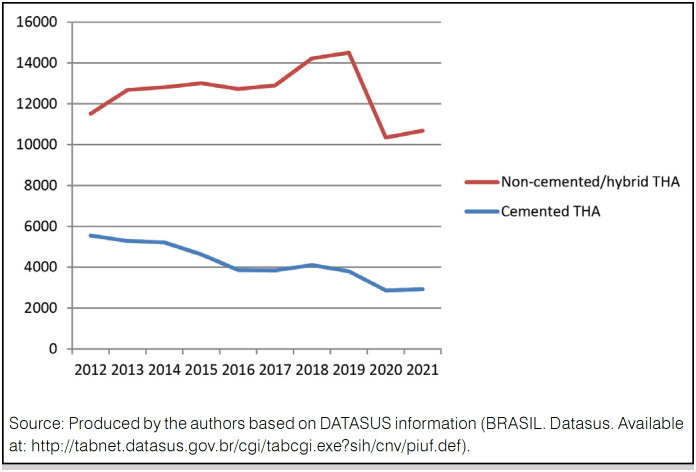
Absolute number of deaths during THA hospitalizations by Brazilian region per year (2012-2021).

The absolute number of deaths in the evaluated period was of 2010 ( Figure 3 ) - the Southeast region had the highest absolute number of deaths (1228, 61.1%) and the North Region had the lowest (22 deaths, 0,01%). Regarding the MR, we observed a lower value in South Region, which presented 1.07 deaths per 1,000 inhabitants; the Southeast Region had the highest MR, reaching 1.88 deaths per 1.000 inhabitants ( Table 5 ).

**Table 5:** Mortality rate (%) during THA hospitalizations by Brazilian region per year (2012-2021).

Region	2012	2013	2014	2015	2016	2017	2018	2019	2020	2021	Average
North	0,88	1,83	2,88	0,94	1,37	0,51	1,55	0,72	0,00	2,56	1,47
Northeast	2,85	2,88	1,65	2,15	1,07	1,53	1,00	1,08	2,25	1,80	1,83
Southeast	2,54	1,98	1,84	1,63	1,70	1,57	1,53	1,79	2,17	2,08	1,88
South	1,18	1,40	1,40	1,01	0,96	0,68	0,77	0,90	1,34	1,04	1,07
Midwest	1,40	0,98	2,49	1,97	1,80	1,12	0,87	1,19	1,37	1,25	1,44
Average	2,14	1,87	1,76	1,52	1,43	1,28	1,20	1,41	1,88	1,74	1,62

Source: Produced by the authors based on DATASUS information (BRASIL. Datasus. Available at: http://tabnet.datasus.gov.br/cgi/tabcgi.exe?sih/cnv/piuf.def ).

## DISCUSSION

The number of primary THA performed in the US in 2014 was 370,770^6^ being projected about 500,000 procedures for the year 2021.^7^ In Brazil, between January 2012 and December 2021, 125,463 procedures were performed under the SUS – the total number of procedures is considerably lower than that projected in the US, because our study did not include the procedures paid privately or funded by health insurance.

Between 2012 and 2019, there was an increase (25.9%) in the number of primary THA performed under the SUS. However, in 2020, the first year in which the COVID-19 pandemic significantly affected the Brazilian health system, there was a significant decrease in the total number of primary THA (28.6%). This decline was even higher when elective procedures (46.3%) were considered - the number of emergency procedures remained stable in the same period ( Figure 2 ),which can be explained by the redefinition of the orthopedic care model during the COVID-19 pandemic, that modified the approach of certain procedures, especially those electives, to provide greater security for patients and health professionals besides to save vital resources, given the reality that was coming.^8^ In the US, in March 2020, 66% of the states have already issued limitation guidelines for elective surgeries. In Poland, there was a drop of 29% to 33% in primary THA numbers between 2019 and 2020.^9^ In Scotland, there was a drop of 53.6%, and after the resumption of elective surgical procedures, Scottish hospitals reached only 40% to 50% of the previous monthly volume.^10^

In the evaluated decade, the total amount spent by the SUS on hospitalizations for primary THA was of 552,218,181.04 BRL. The Southeast Region (the most populous in Brazil) received the largest investment (293,474,673. 20 BRL), corresponding to 53.14% of the total. Proportionally to the number of procedures carried out, there was an increasing trend in spending between the years 2012 to 2019; in the pandemic period (2020-2021) the spending decreased, being lower than the cash spent in 2013 for primary THA hospitalizations ( Table 2 ).

In US (2015), the expenditure associated with THA and total knee arthroplasty (TKA) combined was of 65 billion USD. Between 2003 and 2009, 1.4 to 1.6 billion EUR were spent on primary THA in Germany.^11^ The difference in HC by country, however, can be expressive - in a comparative study between three Canadian hospitals and three US hospitals conducted in 2004, HC averages of US$ 6,766 and US$ 13,339 (p<0,0001), respectively, were found.^12^ In an aggregate study, evaluating 2.8 million THA admissions between 2002 and 2013 in the US, there was an increase in the spent value from 15,792 USD (95% CI, 15,706 to 15,878 USD) in 2002 to 23,650 USD (95% CI, 23,544 to 23,755 USD) in 2013.^13^ In our study, the mean value per hospitalization for primary THA was of 4,394.92 BRL, with an increase of 29.69% in the evaluated period, being lower in the North Region (3,980. 49 BRL) and higher in the Midwest Region (4,820.00 BRL) (Table 3).

Carducci et al. (2020)^14^ found that implant prices were the most dispendious components of total cost across all types of joint arthroplasty, accounting for an average of 53.8% of these expenses and that the increase in hospitalization time would not play a significant role in the value spent to perform these procedures.The amounts paid by the SUS for implants used in cemented THA and non-cemented/hybrid THA may represent, respectively, between 44.14 and 48.06% (1,850.78 to 2,167.42 BRL), and between 66.05 and 69.08% (3,394.38 to 3,887.82 BRL) of the total cost of each hospitalization ( Table 6 ). On the other hand, the literature points out to cost reduction strategies that include reducing the average LOS minimizing preoperative and perioperative risks and investing in postoperative care,^15^ which may constitute alternatives to decrease the burden to the Brazilian public health system.

**Table 6:** Values funded by the SUS for services (hospital and professional) and implant materials used in cemented (SIGTAP CODE 04.08.04.008-4) and non-cemented/hybrid (SIGTAP CODE 04.08.04.009-2) THA. Updated values in September 2022.

Primary Cemented Total Hip Arthroplasty (SIGTAP CODE 04.08.04.008-4)	
Type of Service	Amount Paid per Service
Hospital (5 daily; maximum stay of up until more 2 daily is allowed, ICU daily are funded separately)	1,924.25 BRL
Professional Service	417.46 BRL
Total amount paid for hospitalization expenses	2,341.71 BRL
Implant Materials	Amount paid per implant
Cemented Femoral Modular Component Centralizer (Maximum Quantity: 1)	104.44 BRL
Cement With Antibiotic (Maximum Quantity: 2)	109.62 BRL
Primary/Revision Cemented Acetabular Polyethylene Component (Maximum Quantity: 1)	282.87 BRL
Cephalic Component for Total Hip Arthroplasty (Includes Prosthesis) (Maximum Quantity: 1)	463.48 BRL
Primary Cemented Modular Femoral Component (Maximum Quantity: 1)	1,008.00 BRL
Cephalic Component for Total Hip Arthroplasty (Includes Prosthesis) (Maximum Quantity: 1)	463.48 BRL
Primary Cemented Modular Femoral Component (Maximum Quantity: 1)	1,008.00 BRL
Cemented Charnley type Monoblock Femoral Component (Maximum Quantity: 1)	850.01 BRL
Femoral Cement Restrictor (Maximum Quantity: 1)	28.80 BRL
Cement Without Antibiotic (Maximum Quantity: 2)	60.59 BRL
Total amount paid for implant materials	1,850.78 to 2,167.42 BRL
**Primary Non-Cemented/Hybrid Total Hip Arthroplasty (SIGTAP CODE 04.08.04.009-2)**
**Type of Service**	**Amount Paid per Service**
Hospital (5 daily; maximum stay of up until more 2 daily is allowed, ICU daily are funded separately)	1,924.25 BRL
Professional Service	417.46 BRL
Total amount paid for hospitalization expenses	2,341.71 BRL
Implant Materials	Amount paid per implant
Cemented Femoral Modular Component Centralizer (Maximum Quantity: 1)	104.44 BRL
Cement With Antibiotic (Maximum Quantity: 1)	109.62 BRL
Primary/Revision Metallic Acetabular Component of Biological Fixation (Maximum Quantity: 1)	1,027.28 BRL
Cephalic Component for Total Hip Arthroplasty (Includes Prosthesis) (Maximum Quantity: 1)	463.48 BRL
Primary Cemented Modular Femoral Component (Maximum Quantity: 1)	1,008.00 BRL
Primary Non-Cemented Modular Femoral Component (Maximum Quantity: 1)	1,695.27 BRL
Primary/Revision Polyethylene Acetabular Component for Metallic Component Biological Fixation (Maximum Quantity: 1)	372.78 BRL
3.5 MM Cortical Screw (Maximum Quantity: 3)	15.34 BRL
Acetabular Component Screw (Maximum Quantity: 3)	109.67 BRL
Femoral Cement Restrictor (Maximum Quantity: 1)	28.80 BRL
Cement Without Antibiotic (Maximum Quantity: 1)	60.59 BRL
Total amount paid for implant materials	3,394.38 to 3,887.82 BRL

Source: Produced by the authors based on DATASUS-SIGTAP information (BRASIL. Sigtap. Available at: http://sigtap.datasus.gov.br/tabela-unificada/app/sec/procedimento/publicados/consultar ).

In the US, the average LOS for primary THA decreased from 4.06 to 2.75 days between the years 2002 and 2013,^13^ reaching 2.28 days in 2018, demonstrating a downward trend in that country.^14^ Foote et al. (2009),^16^ analyzing 675 patients submitted to primary THA at a regional hospital in Great Britain, identified an average LOS of 8 days.^16^ Kim et al. (2003),^17^ reviewing the literature on the efficacy of clinical pathways for TKA and THA identified that the standardization of care processes, together with the surgical approach, was associated with a lower average LOS when compared to that observed in patients treated exclusively by surgery. Mertes et al. (2013) demonstrated that integrated care pathways in THA were effective in reducing average LOS (from 6.9 to 5.5 days); elderly and male patients had greater benefits with this strategy.^18^ In our study, the daily average LOS for THA was of 6.8 days; the North Region had the highest average hospital stay (13.1 days) and the South Region presented the lowest average LOS (5.3 days) ( Table 4 ).Factors that are often associated with increased daily LOS include delays or cancellations of surgical procedures, clinical destabilization, nosocomial infection, and waiting for complementary diagnostic tests, vacancies in semi-intensive or intensive care unities or home care – socioeconomic differences may therefore justify the discrepancy observed between distinct Brazilian Regions. The Southeast and South Regions, which have lower average LOS, have higher GDP per capita, while the most deprived regions (North and Northeast) have higher average LOS. However, the increase in hospitalization time observed in North and Northeast Regions did not translate into a proportional increase in HC and MR.

In a study of 10,244 patients undergoing primary THA and TKA for a decade, the perioperative MR was less than 2% in patients under 70 years of age, 4% in patients aged 70 to 79 years, and 21% in patients aged 80 years or older.^19^ In an epidemiological study conducted in the US, which evaluated 2,182,121 primary THA between the years 1998 and 2008, the mean MR was 1.8% or equivalent to 0.44 events per 1,000 days of hospitalization.^20^ In our study, we found an average MR of 1.62% in a decade, slightly below, therefore, of that observed in the literature. The South Region had the lowest (1.07%) average MR and the Southeast Region, the highest (1.88%) ( Table 5 ). The absolute number of deaths in the evaluated period was of 2,010; the Southeast Region presented the highest number (1,228, 61.1%), and the lowest (22 deaths) occurred in the North Region ( Figure 3 ).

The limitations of the current study are in line with other retrospective database studies reviews. Most of them are related to underreporting of cases, lack of information on the socio-demographic characteristics of the affected population, unavailability of specific data concerning underlying hip pathologies, comorbidities and death causes. In addition, we deal with absolute numbers, which does not allow scrutinizing details regarding, for example, to specific expenses with prolonged hospital stay, ICU stay, among other aspects related to primary THA hospitalizations.

## CONCLUSION

The total number of released HAA for primary THA between 2012 and 2021 was of 125,463. The regional distribution occurred as it follows: 65,756 (52%) in Southeast; 33,837 (27%) in South; 14,882 (12%) in Northeast; 9,364 (8%) in Midwest; and 1,624 (1%) in North.

Regarding HC, we detected a total expenditure of 552.218.181,04 BRL in the evaluated period, with the following regional distribution: 293,474,673.20 BRL (53.1%) in Southeast; 144,794,843.11 BRL (26.2%) in South; 61,751,644.36 BRL (11.2%) in Northeast; 45,724,353.80 BRL (8.3%) in Midwest; and 6,472,666.57 BRL (1.2%) in North. The mean value spent by the SUS from 2012 to 2021 per hospitalization was of 4,394.92 BRL - regionally, we observed the expenditure of 4,459.10 BRL in Southeast; 4,280.96 BRL in South; 4,147.19 BRL in Northeast; 4,820.00 BRL in Midwest; and 3,980.49 BRL in North.

The average LOS of the evaluated period was of 6.8 days. Regionally, we observed 6.7 in Southeast; 5.3 in South; 9.2 in Northeast; 7.6 in Midwest; and 13.6 in North.

Regarding the regional distribution, we noted that MR was of 1.88% in Southeast; 1.07% in South; 1.83% in Northeast; 1.44% in Midwest; and 1.47% in North.

After a rise of 25.9% on the number of performed primary THA from 2012 to 2019, there was the impact of the COVID-19 pandemic leading to a reduction of 28.6% from 2019 to 2020, even higher (46.3%) when elective procedures were considered, suggesting the need to evaluate what happened with other elective orthopedic procedures carried out under the SUS in that period.

The data from this study may also assist the government authorities to define appropriate strategies to cope with the socioeconomic impact of the performance of primary THA in the distinct Brazilian Regions.

## References

[1] Jones CA, Voaklander DC, Johnston DW, Suarez-Almazor ME (2000). Health related quality of life outcomes after total hip and knee arthroplasties in a community based population. J Rheumatol.

[2] Vasconcelos KSS, Dias JMD, Dias RC (2006). Relação entre intensidade de dor e capacidade funcional em indivíduos obesos com osteoartrite de joelho. Braz J Phys Ther.

[3] Lehtonen EJ, Stibolt RD, Smith W, Wills B, Pinto MC, McGwin G (2018). Tendências no tratamento cirúrgico das fraturas do colo do fêmur em idosos. Einstein (São Paulo).

[4] Boschin LC, Anacleto OL, Alencar PGC (2003). Artroplastia total de quadril não-cimentada: avaliação radiográfica após seguimento mínimo de 10 anos de pós-operatório. Rev Bras Ortop (São Paulo).

[5] Ferreira MC, Oliveira JCP, Zidan FF, Franciozi CES, Luzo MVM, Abdalla RJ (2018). Artroplastia total de joelho e quadril: a preocupante realidade assistencial do Sistema Único de Saúde brasileiro. Rev Bras Ortop (São Paulo).

[6] Sloan M, Premkumar A, Sheth NP (2018). Projected Volume of Primary Total Joint Arthroplasty in the U.S., 2014 to 2030. J Bone Joint Surg Am.

[7] Singh JA, Yu S, Chen L, Cleveland JD (2019). Rates of Total Joint Replacement in the United States: Future Projections to 2020-2040 Using the National Inpatient Sample. J Rheumatol.

[8] Naito GM, Pimentel CSS, Silva RR, Guedes AAL, Guedes A. (2022). Primary total knee arthroplasties under the Brazilian Public Health Unic System (SUS) - Number of procedures, regional distribution, hospitalization costs, average length of hospital stay and mortality (2012-2021). Res Soc Dev.

[9] Czubak-Wrzosek M, Czubak J, Grzelecki D, Tyrakowski M. (2021). The Effect of the COVID-19 Pandemic on Total Hip and Knee Arthroplasty Surgical Volume in 2020 in Poland. Int J Environ Res Public Health.

[10] Yapp LZ, Clarke JV, Moran M, Simpson AHRW, Scott CEH (2021). National operating volume for primary hip and knee arthroplasty in the COVID-19 era: a study utilizing the Scottish arthroplasty project dataset. Bone Jt Open.

[11] Bleß HH, Kip M. (2017). Weißbuch Gelenkersatz: Versorgungssituation endoprothetischer Hüft- und Knieoperationen in Deutschland.

[12] Antoniou J, Martineau PA, Filion KB, Haider S, Zukor DJ, Huk OL (2004). In-hospital cost of total hip arthroplasty in Canada and the United States. J Bone Joint Surg Am.

[13] Molloy IB, Martin BI, Moschetti WE, Jevsevar DS (2017). Effects of the Length of Stay on the Cost of Total Knee and Total Hip Arthroplasty from 2002 to 2013. J Bone Joint Surg Am.

[14] Carducci MP, Gasbarro G, Menendez ME, Mahendraraj KA, Mattingly DA, Talmo C (2020). Variation in the Cost of Care for Different Types of Joint Arthroplasty. J Bone Joint Surg Am.

[15] Lovald ST, Ong KL, Malkani AL, Lau EC, Schmier JK, Kurtz SM (2014). Complications, mortality, and costs for outpatient and short-stay total knee arthroplasty patients in comparison to standard-stay patients. J Arthroplasty.

[16] Foote J, Panchoo K, Blair P, Bannister G. (2009). Length of stay following primary total hip replacement. Ann R Coll Surg Engl.

[17] Kim S, Losina E, Solomon DH, Wright J, Katz JN (2003). Effectiveness of clinical pathways for total knee and total hip arthroplasty: literature review. J Arthroplasty.

[18] Mertes SC, Raut S, Khanduja V. (2013). Integrated care pathways in lower-limb arthroplasty: are they effective in reducing length of hospital stay?. Int Orthop.

[19] Mantilla CB, Horlocker TT, Schroeder DR, Berry DJ, Brown DL (2002). Frequency of myocardial infarction, pulmonary embolism, deep venous thrombosis, and death following primary hip or knee arthroplasty. Anesthesiology.

[20] Memtsoudis SG, Pumberger M, Ma Y, Chiu YL, Fritsch G, Gerner P (2012). Epidemiology and risk factors for perioperative mortality after total hip and knee arthroplasty. J Orthop Res.

